# LEISURE, LONGITUDINALLY: PARTNER DISAGREEMENTS ABOUT LEISURE DECREASE OVER THE LIFE COURSE

**DOI:** 10.1093/geroni/igz038.2557

**Published:** 2019-11-08

**Authors:** Jill Juris Naar, Shelbie Turner

**Affiliations:** 1 Appalachian State University, Boone, North Carolina, United States; 2 Oregon State University, Corvallis, Oregon, United States

## Abstract

Leisure is a major context within which older couples interact, and researchers have recently called for more longitudinal data analysis exploring how leisure-related couple interactions change over the life course. Several waves of the Midlife in the United States (MIDUS) study include a single-item question asking respondents how much they disagree with their spouse or partner about leisure activities. Given the longitudinal nature of MIDUS, the variable offers great utility to explore shifts in leisure-related couple interactions over the life course. Utilizing longitudinal data from Wave 1 (1995-1997), 2 (2004-2006), and 3 (2013-2015) of the MIDUS study, we explored how leisure-related partner disagreement changed with increased age (age range = 20-93). We first ran an unconditional multilevel model, which revealed that 68% of the variation in leisure-related spousal disagreement was attributed to within-person differences over time, justifying our analysis of longitudinal within-person change. An age-based growth curve model then revealed that leisure-related partner disagreements decreased linearly over the life course (Estimate = -0.01, SE = 0.001, p<.0001). Men reported more leisure-related partner disagreements than women at age 20 (p = 0.002). But men’s reported disagreements decreased over the life course at a faster rate than did women’s reported disagreements (p = 0.03), so that from ages 70-93, men reported less disagreements than women. To our knowledge, this is the first longitudinal study to explore leisure-related couple disagreements over an extended period of time (20 years). The significance of our results sheds light on the value of longitudinal research on leisure.

